# Exploration of oral hygiene practices, oral health status, and related quality of life of individuals residing in the Rorya district of Tanzania, East Africa

**DOI:** 10.3389/froh.2024.1435555

**Published:** 2024-10-01

**Authors:** Priyanka Gudsoorkar, Rachael Nolan, Sweta Kafle, Aayush Dubey

**Affiliations:** College of Medicine, University of Cincinnati, Cincinnati, OH, United States

**Keywords:** oral health, social determinants of health, East Africa, dental caries, periodontitis, oral health-related quality of life, mixed-methods

## Abstract

**Introduction:**

Oral health substantially impacts individuals’ quality of life, making it an important target for global health interventions. This research describes oral health status, practices, and beliefs within the Rorya district of Tanzania to understand barriers to care.

**Methods:**

To quantify physical oral health status, intraoral examinations were conducted on adults, noting the Decayed Missing and Filled Teeth (DMFT) and Comprehensive Periodontal Inflammatory Burden Index (CPBI). Oral Health-Related Quality of Life (OHRQoL) and semi-structured interviews were conducted to understand oral hygiene behaviors and beliefs. Data was analyzed via two-sample *t*-tests, Pearson's statistics, and NVIVO.

**Results:**

A purposive sample (*n* = 139) of participants self-reported to reside in either Burere (*n* = 32), Nyambogo (*n* = 52), or Roche (*n* = 55) were assessed. A two-sample *t*-test revealed females (*n* = 67; x¯ = 7.1; SD = 5.4; *p* < 0.05) have a significantly higher DMFT score than males (*n* = 72; x¯ = 3.7; SD = 3.9). Moreover, the OHRQoL score of females (*n* = 67; x¯ = 12.10; SD = 14; *p* < 0.05) were significantly higher than males (*n* = 72; x¯ = 10.16; SD = 3). In contrast, males have significantly higher CPBI scores (x¯ = 3.8; SD = 1.5; *p*=<0.05) than females (x¯ = 3.0; SD = 1.3). Additionally, older age groups presented higher GI and PISA scores, while the younger group (20–30 years) displayed the highest mean DMFT score. The themes that emerged from semi-structured interviews were “pearls of laughter guarded by wisdom teeth,” “whispered tales of oral tides and communal echoes,” and “tales of the tooth fairy.”

**Discussion:**

In this community, proper oral health maintenance techniques are vital yet frequently disregarded, mainly due to disparities in access to resources, reflected in oral health scores. Addressing this is a crucial intervention, presenting an opportunity to uplift overall well-being. Moreover, gender and age disparities in oral health highlight the urgent need for tailored interventions.

## Introduction

Oral diseases present an escalating public health predicament worldwide. Despite being largely preventable and non-communicable, untreated dental caries, periodontal diseases, tooth loss, and oral cancers significantly contribute to the overall global health burden (1–3). At an individual level, untreated oral conditions—pain, tooth loss, cavities, and reduced functional ability—markedly diminish one's quality of life. Furthermore, robust evidence underscores the bidirectional link between oral and systemic health (4–6). Periodontal diseases are associated with a spectrum of chronic conditions, including diabetes and cardiovascular diseases (6–9). Subsequently, the World Health Organization (WHO) underscores the importance of oral health as a definitive indicator of total health and social interaction (10, 11).

Dental caries is the most common global disease in developing regions, including East Africa (3, 12). The increased prevalence of dental caries is attributed to inadequate dental hygiene, restricted access to dental services, and insufficient awareness of caries’ risks, all compounded by sugar-rich diets (13–15). Additionally, the high prevalence of Cancrum oris or “noma,” a severe oral disease in these regions, exemplifies the dire consequences of inadequate oral hygiene, poverty, malnutrition, and compromised immune systems (16, 17).

Among marginalized and low-resource populations in developing nations, dental decay remains predominantly untreated, signaling a significant burden of oral health-related morbidity and mortality (18–20). Contributory factors such as limited access, a deficit of dental professionals, other barriers to care, and cultural misinformation on oral health practices amplify poor health outcomes. These disparities emphasize the need for integrated oral healthcare and broader public health strategies to mitigate the global disease burden.

Primary prevention strategies for plaque control, such as regular brushing with fluoridated toothpaste and adopting a twice-daily brushing habit, serve as the cornerstone of oral disease prevention (21–23). In locales with low access to oral healthcare and resources, the value of oral health education becomes vital. Literary evidence consistently reinforces this, suggesting that educational interventions can markedly elevate oral health knowledge, transform attitudes, and refine practices, culminating in decreased prevalence and intensity of oral diseases.

In Tanzania, where oral health challenges are particularly acute, epidemiological investigations highlight that the improper use of “miswak” or chewing sticks is a key factor exacerbating oral health issues, including plaque accumulation, calculus formation, increased periodontal diseases, tooth loss, and decay (24–27). Additional practices such as sharing toothbrushes, prompted by the limited availability of modern oral hygiene instruments, introduce further hazards by their association with the spread of infectious diseases and stress the need for effective oral hygiene practices (28).

This study aims to gain perspective on oral health practices, cultural awareness, and access to oral health aids by collecting brushing narratives from residents in the Rorya district of Tanzania, East Africa. These narratives and community-based focus groups will help further elucidate the oral hygiene beliefs, knowledge, and traditions within the Rorya district, Tanzania.

Because the quantification of Oral Health-Related Quality of Life (OHRQoL) has emerged as a central focus within this research, this study uses OHRQoL as a comprehensive construct to integrate the subjective evaluation of one's oral health on life quality, including functional and emotional well-being, expectations, care satisfaction, and self-perception (29, 30). Additionally, the Decayed Missing Filled Teeth (DMFT) index and Comprehensive Periodontal Inflammatory Burden Index (CPIBI) are used to assess the impact and prevalence of oral disease and dental caries among the adult population, providing a comprehensive understanding of oral health status. As such, this study represents a significant opportunity to develop interventions to improve oral health outcomes in these communities. This research was approved by UC protocol FWA#003152.

## Methods

The sample (*n* = 139) consisted of individuals residing in the Rorya district of Tanzania, East Africa, recruited via purposive sampling to match the study's focus. Eligibility criteria required participants to be adults (≥18 years) who were residents of the Burere, Nyambogo, and Roche in the Rorya district of Tanzania and conversant in Swahili or English. Anonymity with informed consent was preserved using a unique 6-digit code indicating data collection specifics. For the quantitative measures, a single administration of the validated oral health-related quality of life (OHRQoL) instrument (30) was used at baseline, and oral examinations were conducted to assess the decayed, missing, and filled teeth (DMFT) index (See Appendix Attachment 1). A portable dental chair and lighting apparatus aided all assessments. The Comprehensive Periodontal Inflammatory Burden Index (CPIBI)—including gingival index (GI), bleeding on probing (BOP), and periodontal inflamed surface area (PISA) assessments were also used. A semi-structured interview guide was used to conduct audio-recorded interviews and focus groups for the qualitative measures. To guarantee cultural and linguistic appropriateness, English-Swahili bilingual interpreters conducted interviews and focus groups in collaboration with University of Cincinnati (UC) affiliates and volunteer local oral health program members. Data were transcribed verbatim and stored on a secure, password-protected PC in a locked office, only accessible within UC premises.

Quantitative analysis was performed on cleaned data (*n* = 139) after excluding incomplete records. Statistical Analysis Software (SAS) 9.4 facilitated DMFT, GI, BOP, PISA, and CPIBI metrics calculations. Pearson's correlation and logistic regression models assessed inter-index associations and gender-based prediction, respectively, while paired *t*-tests examined age-index correlations. Qualitative data from interviews *(n* = 39) underwent descriptive coding (32) by dual coders—one employing manual techniques, the other NVivo 14.0 (33). Codes were consolidated into clusters to extract themes aligned with the research objectives.

All data were encrypted and disassociated from personal identifiers. The synthesis of interviews, focus groups, and quantitative assessments underpinned the development of a context-sensitive oral health promotion initiative in Tanzania. Participants received oral health kits and bilingual brushing instructions as compensation.

## Results

### Participants and demographics

A purposive sample (*n* = 139) of villagers self-reported to reside in either Burere (*n* = 32), Nyambogo (*n* = 52), or Roche (*n* = 55). Most participants identified as male (*n* = 72; 51.79%), with a mean age (x¯ = 42.38; *SD* ± 16.91) years (See Table 1).

**Table 1:** Mean and standard deviation for DMFT + and CPIBI^a^.

		No. (%)	Decayed mean (*SD*)	Missing mean (*SD*)	Filled mean *(SD*)	DMFT mean (*SD*)	CPIBI mean (*SD*)
Sample		139	3.8 (4.4)	1.5 (2.5)	0 (0)	5.2 (5)	5.5 (1.4)
Sex	Males	72 (51.8)	2.6 (3.4)	1.1 (1.9)	0 (0)	3.7 (3.9)	6.8 (1.5)
	Females	67 (48.2)	5.1 (4.9)	1.9 (2.9)	0 (0.1)	7.1 (5.4)	4.0 (1.3)
Age groups	10–20	10 (7.2)	4.1 (5.1)	1.2 (2.5)	0 (0)	5.3 (5)	3 (1.3)
	20–30	32 (23)	5.5 (5)	0.9 (1.2)	0 (0)	6.4 (5.5)	3.3 (1.5)
	30–40	32 (23)	3 (3.8)	0.9 (1.4)	0 (0.2)	4 (3.9)	2.9 (1.3)
	40–50	17 (12.9)	3.8 (4)	1.1 (2.5)	0 (0)	5 (4.7)	4 (1.4)
	50–60	24 (17.3)	3.0 (3.2)	1.5 (2.5)	0 (0)	4.5 (4.4)	3.8 (1.2)
	60+	24 (17.3)	3 (4.7)	3.5 (3.6)	0 (0)	6.5 (5.9)	4 (1.1)

+DMFT [Decayed (D) + Missing (M) + Filled (F) Teeth].

^a^ CPIBI, comprehensive periodontal inflammatory burden index.

### DMFT

An average DMFT score of x¯ = 5.2 (*SD* ± 5; min to max = 0–24) with higher scores indicating a greater prevalence of dental caries was observed. The statistical analysis delineated a gender disparity in oral health with females (*p* < .001) (x¯ = 7.1; *SD* ± 5.4) who self-reported significantly higher DMFT scores when compared to males (x¯ = 3.7; *SD* ± 3.9), implying a higher burden of dental caries among females. Regression analysis showed that age was not a strong predictor of DMFT score. However, the y-intercept (*p* < .001) (coefficient = 3.13; *SD* ± 1.4) for sex implied that other unmeasured variables could be contributing to gender differences in DMFT scores, meriting further investigation.

### CPIBI

The sample had an average CPIBI score of x¯ = 5.5 (*SD* ± 1.41; min to max = 1–7), with higher scores indicating poor periodontal health. Results from the two-sample *t*-test suggested moderate periodontal inflammation, with males who self-reported significantly higher CPBI scores (x¯ = 6.8; *SD* ± 1.5; *p* = 0.004) when compared to females (x¯ = 4.0; *SD* ± 1.3). Regression analysis showed no significant association between CPIBI and demographics.

### GI

An average gingival score of x¯ = 0.98 (SD = 0.90; min to max = 0–3) with higher scores indicating poor gingival health was observed. These results indicated mild gingival inflammation in the sample, although a two-sample *t*-test revealed no significant difference in average GI scores in males (x¯ = 1.1; SD = 0.9; *p* = 0.058) compared to females (x¯ = 0.8; *SD* = 0.9). No correlation between GI and sex was found. However, regression analysis showed a moderate positive linear association between GI index and age (*p* = 0.001), with age explaining 7.53%of GI variance in the sample. Both sex (*p* < 0.001) and age (*p* = 0.001) were found to be statistically significant predictors of GI, which underscored the importance of considering biological factors, among others, when evaluating gingival health across groups.

### OHRQoL

A mean OHRQoL score x¯ = 11.10 (*SD* ± 4.72; min to max = 3–18, with higher scores indicating decreased quality of life was observed. The two-sample *t*-test demonstrated that females had significantly decreased oral health-related quality of life (*n* = 67; x¯ = 12.10; *SD* ± 1.4; *p* ≦  0.05) when compared to males (*n* = 72; x¯ = 10.16; *SD* ± 3)

### Qualitative results

Of the sample, only 36 (*n* = 139) participated in the qualitative portion of the study.

After developing code clusters, two independent coders compared findings to build themes that aligned with the aims of the present study. Using a constant comparison method, six themes were developed. Each theme provided information on oral hygiene practices and factors related to oral health-related quality of life and was identified as (1) Whispered tales of oral tides and communal echoes, defined as the household dynamics and social influences on oral health behaviors; (2) Pearls of laughter guarded by wisdom teeth, defined as the oral health-related knowledge, attitudes, beliefs, and practices that influence oral health hygiene; and (3) Tales of the tooth fairy, defined as the lifetime of oral health practices that have led to poor oral health-related quality of life (See Table 2).

**Table 2:** Thematic definitions.

Themes	Definition
Whispered tales of oral tides and communal echoes	This theme captured the composition, structure, and interactions between household family members and their community concerning oral health behaviors
Pearls of laughter guarded by wisdom teeth	This theme explored the knowledge, attitudes, beliefs and cultural practices related to oral health hygiene
Tales of the tooth fairy	This theme focused on the lifetime of self-reported oral hygiene practices that have led to poor oral health and reduced quality of life

Quantitative results. All (*n* = 139) villagers participated in the quantitative portion of the study.

### Themes

The first theme, “whispered tales of oral tides and communal echoes,” depicted the sample's household dynamics and various social influences affecting individual oral health. Within the familial structure of the Rorya district, a clear delineation existed in oral hygiene behaviors across age and economic lines. Self-reported information on the number of villagers in each household revealed that the average number of persons in residence was seven. Each household, on average, consisted of four adults and three children. Brushing frequencies (*n* = 39) were reported to be once a day (*n* = 9), twice a day (*n* = 5), and three times a day (*n* = 3), with select oral hygiene tools aplastic toothbrushes (*n* = 12), “miswak” sticks (*n =* 21), or both (*n* = 18)—reflecting availability, personal choice, and gender-roles. The following quote best exemplified this theme by stating, “*[My] children and husband have their toothbrushes, and I just use anything, but everybody uses toothpaste* (38-year-old female; mother of 6 children).” Villagers (*n* = 24) reported that children brushed their teeth, and the frequency of brushing their teeth was once a day (*n* = 6) and twice every day (*n* = 2), respectively. Familial income and level of adult supervision were found to be leading factors of intermittent toothpaste usage among children, with one participant (42-year-old male; parent of 5 children) noting, “*They are sometimes using [toothpaste]. Sometimes they don't have money,*” and another who stated, “*Children are not using it [toothpaste] because I fear they might swallow the toothpaste.*”

Social factors in the home, such as toothbrushing location, HIV awareness, and smoking, also influenced teeth-brushing behaviors. For example, mostly (*n* = 39) individuals reported brushing their teeth outside the house. In contrast, only one individual reported brushing their teeth inside the home (*n* = 1). The additional social factor, health of post-HIV awareness, demonstrated that some villagers (*n* = 9) understood the risks associated with sharing personal hygiene items, with one participant (36-year-old male) who noted, “*Since this HIV comes, no one now accepts to share it [toothbrush].*” The habit of smoking (*n* = 22) and secondhand smoke exposure (*n* = 19), which also included inhalation of cooking fire or burning charcoal (*n* = 16) and wood chips and sticks (*n* = 16), appeared to have a strong influence on oral hygiene. Extrapolating from the household to the societal level, environmental determinants such as smoke from cooking fires were shown to be an everyday occurrence, with one villager (62-year-old female) who stated, “*Yes, whenever we are cooking, we are inhaling fumes that are good for teeth pain.*” This concurrent evidence implies oral health behaviors and culture are inextricably linked and reflect the social context of a villager's family and communal influence.

The second theme, “pearls of laughter guarded by wisdom teeth,” sheds further light on the ingrained oral health knowledge, attitudes, beliefs, and culture in the Rorya District that dictate oral health hygiene. This theme also included perceived indicators of cleanliness, rationales for oral hygiene habits, and using and acquiring alternative oral hygiene tools. In the study, villagers perceived benchmarks of oral cleanliness brushing (*n* = 32), breathing with no bad breath (*n* = 5), observing one's teeth in the mirror with no evidence of food-related matter stuck in teeth (*n* = 3), and uncertain (*n* = 2). Participants stated that concerning the timing of tooth brushing, morning (*n* = 20) was preferred, with variable motivations to brush throughout the day caused by removing food remnants (*n* = 4), the pursuit of a fresh feeling (*n* = 4), and out of sheer habit (*n* = 5). For example, one participant (32 age, female) said, “*Morning is the best time to brush your teeth when you wake up and have food particles in your mouth; Sometimes, I use sticks to brush my teeth; when my teeth ache, as it makes me feel better*.”

Resourcefulness and adaptation of villagers in this area are made evident by the use of adaptive tools for tooth brushing, such as sticks (*n* = 15), fingers (*n* = 4), and salt (*n* = 1). The necessity for a new tool, however, was based on the physical condition of the tool, with general wear and tear (*n* = 8) and the desire to replace the tool daily. For individuals who reported using a plastic toothbrush, the toothbrush was changed due to bristles coming off (*n* = 4), bristles getting hard (*n* = 4), and the toothbrush no longer cleaning as expected (*n* = 1). Concerning the Roya District community, hygiene practices reveal a tapestry of techniques, toothpaste brands, and water sources, further depicting adaptation to available resources. Toothpaste brands such as Colgate® (*n* = 12) and Whitedent® (*n* = 11) are reported to be inconsistently used in the area, depending on availability. Diverse toothbrushing techniques reported by the sample included moving the toothbrush from front to back (*n* = 22) and a combination of styles that could be more directionally specific (*n* = 8). Use of water after brushing was noted (*n* = 28), with many villagers sourcing their water from a well (*n* = 14), rainwater (*n* = 11), dam-stored water (*n* = 10), and lake/river water (*n* = 9). For storage of their toothbrushing tools, various locations are noted, such as in a cup (*n* = 13), on the roof of their residence (*n* = 10), in a pocket/pouch (*n* = 2), embedded in the wall (*n* = 1), in a box (*n* = 1), or a basket (*n* = 1). Time spent brushing teeth in the sample was most notably influenced by the daily rhythm of life, including chores such as farming and fetching water, which made the time spent brushing teeth from a reported 1–2 min to several hours. Thus, oral health maintenance was not perceived as a stand-alone task but was often woven into daily life, highlighting the dynamic nature of health practices in this underserved community.

The final and third theme, “Tales of the Tooth Fairy,” chronicled the oral health history of villagers within the Rorya district and captured the genesis and evolution of dental hygiene practices and oral ailments affecting the community. It also revealed that the typical age of onset for teeth brushing, and transmission of hygiene knowledge was 10 years for adults in the sample. However, the onset of toothbrushing for these adult children was reported to be 5 years of age. Villagers most commonly received oral hygiene education through observation and instruction from various sources (*n* = 24), school (*n* = 15), their mother (*n* = 8), father (*n* = 3), both parents (*n* = 8), or someone else (*n* = 1). As one participant (female sex) stated, “*I just saw my brother and father brushing their teeth.*” *F*rom this experience, she asked if she could have a toothbrush to start brushing her teeth. Yet, this education was eclipsed by the volume of oral health challenges expected in the community and the cumulative effect of untreated oral health and disease. Villagers in the sample reported multiple dental problems such as chronic pain (*n* = 19), holes in their teeth (*n* = 15), swelling (*n* = 6), difficulty chewing and bleeding (*n* = 10), gum issues (*n* = 4), teeth sensitivity (*n* = 2), bad odor (*n* = 2), teeth mobility (*n* = 1), and cracks in teeth (*n* = 1). For example, one villager (female sex) mentioned, “*My teeth hurt; some of them have holes, and that leads to swelling in the mouth.*” These individuals also reported a firm reliance on local remedies and herbs (*n* = 9), toothpaste mixed with soot or salt (*n* = 12), sticks (*n* = 11), and additional unconventional treatments such as paper and petrol (*n* = 5). Due to the prevalence of dental issues, a segment of the community sought medical intervention, with several seeking hospital (*n* = 11) and pharmaceutical services (*n* = 7). However, a notable portion never sought nor received medical or dental care (*n* = 16).

## Discussion

This research substantiated the pivotal role of oral health as a significant determinant of well-being. It echoed previous findings highlighting the burden of oral disease and high DMFT scores in East Africa and Tanzania (32, 33). The present findings also implicated multifaceted factors that have contributed to the high prevalence of dental caries in the region, such as the scant access to oral health and near-zero-filled teeth subindex, despite concerns about tooth decay. These findings emphasized a flawed pattern of oral care where villagers only sought dental-type services in dire circumstances that often resulted in extractions rather than preventive or restorative treatments.

Socioeconomic constraints also profoundly affected the burden of oral disease in the region, with the “all or nothing” access to toothbrushes and fluoridated toothpaste significantly contributing to the lack of preventive care (31). Therefore, villagers in the Rorya District used more indigenous methods like chewing sticks, which, despite their potential effectiveness to remove plaque, were not sufficient oral hygiene tools due to the absence of fluoridation and the likelihood of abrasion caused by prolonged use (34, 35). Gender disparities in this region were also prevalent, with women exhibiting higher DMFT scores. In contrast, males showed higher CPIBI scores and higher rates of gingival disease, which may be linked to increased exposure to environmental factors such as secondhand smoke (36, 37). Gender biases in resource access and cultural practices were also documented and likely influenced the adoption of oral health hygiene behaviors between men and women. For instance, females in the sample often preferred miswak over toothbrushes. These differentiated practices, alongside late tooth brushing initiation and suboptimal dietary habits, placed villagers in the region at significant risk for oral cancer, among other issues attributing to decreased quality of life.

Aside from gender, older age groups in the sample reported higher GI and PISA scores, which previously have been shown to increase the likelihood of developing systemic disorders such as diabetes and cardiovascular disease, making strategies and interventions that address both underlying chronic conditions and oral health vital in this region (4). Targeted treatments and sustainable preventive initiatives have become essential in this region to improve oral health outcomes and contribute to broader well-being objectives such as health equity, education, and socio-economic advancement (38, 39).

## Conclusion

This study has provided a nuanced understanding of the oral health landscape in the Rorya district and stressed the salient connection between life quality and oral hygiene as integral components of functionality.

Our findings revealed that the intersection of cultural practices, inadequate resources, and lack of oral health knowledge has culminated in a significant dental burden in the Rorya District of Tanzania, East Africa. Disparities found, most notable among women and younger demographics with higher mean DMFT scores, have challenged the notion that oral health issues predominantly affect older populations. Future efforts should prioritize delivering treatment to these villages to address barriers related to accessibility and availability.

## Data Availability

The raw data supporting the conclusions of this article will be made available by the authors, without undue reservation.

## Appendix

Appendix Attachment 1 Oral health-related quality of life (OHRQoL).

Instrument: oral health-related quality of life (31).

Dimensions measured: daily activities, social activities, conversation.

Response format: 6-point Likert scale “all of the time” to “none of the time.”

1. Have problems with your teeth or gums affected your daily activities, such as missing school, college, or work?

**Table:**

All of the time 6	5	4	3	2	None of the time 1

2. Have problems with your teeth or gums affected your social activities, such as family gathering, meeting with friends, playing outdoors?

**Table:**

All of the time 6	5	4	3	2	None of the time 1

3. Have problems with your teeth or gums affected your conversations with friends, family, or work colleagues?

**Table:**

All of the time 6	5	4	3	2	None of the time 1
